# Age-associated mRNA expression changes in bovine endometrial cells in vitro

**DOI:** 10.1186/s12958-017-0284-z

**Published:** 2017-08-14

**Authors:** Nao Tanikawa, Ayaka Ohtsu, Ryouka Kawahara-Miki, Koji Kimura, Shuichi Matsuyama, Hisataka Iwata, Takehito Kuwayama, Koumei Shirasuna

**Affiliations:** 1 Laboratory of Animal Reproduction, Department of Animal Science, Tokyo University of Agriculture, 1737 Funako, Atsugi, Kanagawa 243-0034 Japan; 2 NODAI Genome Research Center, Tokyo University of Agriculture, Setagaya, Tokyo, 156-8502 Japan; 3 Laboratory of Reproductive Physiology, Graduate School of Environmental and Life Science, Okayama University, Tsushima, Okayama, Japan; 4 Animal Feeding and Management Research Division, National Institute of Livestock and Grassland Science, Nasushiobara, Tochigi, Japan

**Keywords:** Uterus, Aging, Inflammation, Interferon tau, Cow

## Abstract

**Background:**

Endometrial cells secrete various cytokines and the dysfunction of endometrial cells may directly lead to infertility. Interferon tau (IFNT) secreted by trophoblast cells, a well-known pregnancy recognition signal in ruminants, acts on the uterus to prepare for pregnancy. Aging causes cellular and organ dysfunction, and advanced maternal age is associated with reduced fertility. However, few studies have investigated age-dependent changes in the uterus.

**Methods:**

Using next generation sequencing and real-time PCR, we examined mRNA expression in bovine endometrial cells in vitro obtained from young (mean 45.2 months) and aged (mean 173.5 months) animals and the effects of IFNT depending on the age.

**Results:**

We showed that inflammation-related (predicted molecules are *IL1A*, *C1Qs*, *DDX58*, *NFKB*, and *CCL5*) and interferon-signaling (predicted molecules are *IRFs*, *IFITs*, *STATs*, and *IFNs*) pathways were activated in endometrial cells obtained from aged compared to young cows. Also, the activation of “DNA damage checkpoint regulation” and the inhibition of “mitotic mechanisms” in endometrial cells obtained from aged cows were evident. Moreover, we showed lower cell viability levels in endometrial cells obtained from aged compared to young cows. Although treatment with IFNT upregulated various types of interferon stimulated genes both in endometrial cells obtained from young and aged cows, the rate of increase by IFNT stimulus was obviously lower in endometrial cells obtained from aged compared to young cows.

**Conclusions:**

Endometrial cells obtained from aged cows exhibited higher levels of inflammatory- and IFN-signaling, and dysfunction of cell division compared with young cows. In addition, a high basal level of IFN-related genes in endometrial cells of aged cows is suggested a concept of “inflammaging”.

**Electronic supplementary material:**

The online version of this article (doi:10.1186/s12958-017-0284-z) contains supplementary material, which is available to authorized users.

## Background

The uterus is the essential organ for pregnancy that provides the platform for embryo development, elongation, implantation, placentation, and fetal development in ruminants. In cattle, although the fertilization rate is estimated to be about 90% by artificial insemination, the majority of embryonic losses occur between 8 and 16 days after insemination [1], indicating the importance of the duration within the uterus. When pregnancy is established, interferon tau (IFNT), a well-known pregnancy recognition signal in ruminants [2, 3], is secreted by embryonic trophoblast cells between days 10 to 25 of pregnancy [4]. IFNT acts to prepare the body for pregnancy, such as the inhibition of luteolytic action, induction of immune modulation, promotion of embryonic growth, and implantation of the conceptus [5]. On the other hand, it is becoming increasingly accepted that many normal reproductive processes display hallmark signs of inflammation, including ovulation, implantation, and parturition [6]. In addition, pro-inflammatory cytokines produced by the embryo as well as endometrial tissue play key roles in physiological inflammatory phenomena, including implantation; therefore, uncontrolled and/or pathological situations such as an infection or disease in the uterus may directly lead to infertility [6, 7].

Aging is the result of complex interactions causing dysfunctions in cells and organs. Recently, it has been widely recognized that physiological or pathophysiological aging can be driven by pro-inflammatory cytokines [8–11]. The plasma concentrations for various pro-inflammatory cytokines, including interleukin-1β (IL1B), IL6, and exhibit age-dependent increases in healthy humans [12], suggesting the emerging concept of “inflammaging” as an age-dependent low-grade, chronic, and systemic inflammatory state [10].

In terms of reproduction, it is well known that advanced maternal age is associated with reduced fertility and adverse pregnancy outcomes, and the age-related decline in oocyte quality is well understood [13, 14]. We recently suggested that the oviduct senescence was dependent on the aging of cows because aged oviduct epithelial cells exhibited higher levels of inflammatory cytokines, excessive reactive oxygen species production, and decreased villous and proliferative activities compared to young oviduct epithelial cells in cattle [15]. In terms of uterine function, the uterine dysfunction and senescence and contractile activity in the myometrium in young rats was significantly greater than that in aged rats [16]. Moreover, immune- and inflammatory response-related pathways were upregulated depending on the age of rat uterine horn as shown by microarray analysis [16]. In addition, Hirota et al. [17] demonstrated that uterine-specific p53 deficiency conferred uterine senescence, resulting in the promotion of preterm birth in mice. However, there have been few investigations on the age-dependent functional changes in the uterus.

In the present study, we hypothesized that bovine endometrial cells exhibit age dependent changes in their characteristics and functions, and thus an investigation of this system could be a powerful tool for understanding uterine aging. We further hypothesized that the influence of IFNT on endometrial cells will change as cows age, resulting in difficulties in the induction of appropriate changes for pregnancy by IFNT. Therefore, we examined intrinsic changes in the mRNA expression patterns depending on aging and the effect of IFNT between endometrial cells obtained from young and aged cows by next generation sequencing.

## Methods

### Collection of bovine uterine tissue

Japanese Black cow uteri were collected from a local slaughterhouse within 10–20 min of exsanguination. The stage of the estrous cycle was determined based on macroscopic observations of the ovary and uteri, and healthy uterus were selected [18–20]. Uterine tissues ipsilateral to the corpus luteum were collected at days 10–15 of the estrous cycle because during this phase embryo elongation and IFNT secretion from trophoblast cells within the uterus occurs. Tissue samples were transported to the laboratory (ice cold phosphate-buffered saline (PBS) containing antibiotics), and utilized for cell culture. In the present study, we defined cows aged between 150 and 210 months as old (mean 173.5 months), and cows aged between 28 and 68 months as young (mean 45.2 months). Our in vitro experiment used 8 young and 12 aged uterine tissues.

### Isolation of endometrial cells

Endometrial cells were isolated from uterine tissues as described previously [21] because they showed that IFNT clearly stimulated ISGs expression using mixed culture system between epithelial and stromal cells. Briefly, uterine horns were cut and then washed with PBS containing amphotericin B (1 μg/mL) and gentamicin (0.5 μg/mL) (Sigma-Aldrich, St Louis, MO). The uterine lumen was flushed slowly with PBS. Endometrium tissues, including epithelial and stromal cells (without uterine muscle layers), were cut to 5 mm in size. These tissues were collected and incubated in 0.1% collagenase solution (Roche Diagnostics, Mannheim, Germany) in Dulbecco’s modified Eagle’s medium/F-12 (DMEM/F-12; Life Technologies, Carlsbad, CA) for 60 min at 37 °C with gentle stirring. After centrifugation at 500 g × 10 min at room temperature, the pellet was resuspended and filtered (70 μm). The cells were washed and treated with erythrocyte-lysing buffer (BD Bioscience, Franklin Lakes, NJ) and resuspended in DMEM/F-12 supplemented with 5% fetal calf serum (FCS; ICN, Costa Mesa, CA), amphotericin B (1 μg/mL), and gentamicin (0.5 μg/mL). The cells were plated and cultured at a concentration of 1 × 10^5^cells/well in 24- or 48-well culture plates (Thermo Fisher Scientific, Waltham, MA). As the endometrial cells, including epithelial and stromal cells, attached within 48 h after plating, the culture media was changed and cells were further cultured for another 24 h. The isolated endometrial cells were not pooled and cultured separately for each individual cow. Using fluorescence immunocytochemistry of cytokeratin (a marker of epithelial cells) and vimentin (a maker of stromal cells), we confirmed existence of cytokeratin- and vimentin-positive cells in mixed culture model (data not shown). In addition, we confirmed the mRNA expression of markers such as epithelial cells (e.g. KRT18, KRT19) and stromal cells (e.g. vimentin, desmin, MMP1) in this culture model and the expressions of these markers (RPKM value) did not differ between endometrial cells obtained from young and aged cows (data not shown).

### Transcriptome analysis of endometrial cells

For transcriptome analysis, we used endometrial cells obtained from 4 young and 5 aged cows, respectively. After cultured for 24 h, the cells were washed twice in PBS and treated in the absence (as a control) or presence of IFNT (1 ng/mL) in DMEM/F-12 supplemented with antibiotics for 24 h because we reported this dose of IFNT clearly stimulated ISGs expression in bovine cells [22]. Recombinant bovine IFNT was gifted by Professor RM Roberts (bTP-509A, University of Missouri, Columbia) [2]. The activity of IFNT was then determined by a viral resistance assay using bovine kidney MDBK cells and was found to be 59,050 IU/mL (5.95 × 10^5^ IU/mg at 456 μM). After incubation, the cells were washed twice with PBS and total RNA was extracted using an RNAqueous RNA Isolation Kit (Thermo Fisher Scientific). In this next generation sequencing analysis, RNA samples were pooled in each group. After assessing the RNA quality (RIN value 7.0 or more) using a 2100 Bioanalyzer (Agilent Technologies, Palo Alto, CA, USA), libraries were prepared with a TruSeq RNA Sample Preparation Kit (Illumina; San Diego, CA). Using these libraries, clusters were generated with an Illumina cBot and one lane for the two groups were sequenced as 100-base reads (single end) by an Illumina HiSeq Sequencing System. Image analysis, base calling, and quality filtering were performed with CASAVA ver. 1.8.4 (Illumina) according to the manufacturer’s instructions (DDBJ Sequence Read Archive, DRA005722). Derived sequence data were aligned with the bovine genome sequence (UMD3) to count the sequence reads using CLC Genomics Workbench (Qiagen; Redwood City, CA). To predict upstream transcriptional regulators, significantly differentially expressed genes were analyzed using the Canonical Pathways, Upstream Regulator function, and Diseases and Bio Functions in Ingenuity Pathways Analysis (IPA) program (Qiagen). IPA was used to identify biofunctional signaling networks associated with differential changes in mRNA expression in young or aged bovine endometrial cells. Kal’s z-test was used to analyse gene set enrichment in the functional categories. The data used for this analysis comprised a gene list of 24,596. The data were further filtered with IPA at an FC threshold of 2.0. and FDR adjusted *p*-value <0.05.

### Cell viability assay

Cell viability was assessed by the WST1 assay kit (Roche, Mannheim, Germany) according to the manufacturer’s instructions. To analyze the viability of endometrial cells, we used endometrial cells obtained from 5 young and 7 aged cows, and these were isolated as follows. Briefly, isolated endometrial cells were incubated at 1 × 10^4^ cells/well in 96-well plates in DMEM/F-12 supplemented with 5% FCS and antibiotics for 48 h. Then, the cells were washed twice in PBS and further incubated in DMEM/F-12 supplemented with antibiotics for 24, 48, or 72 h. After incubation, the cells were reacted by WST1 solution for 3 h, absorbance at 450 nm was measured using a microplate spectrophotometer (DS Pharma Biomedical Co, Ltd).

### Real-time RT-PCR

**Fig. 1:**
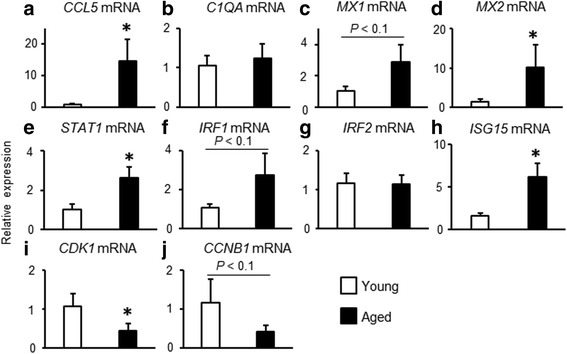
Age-dependent changes in mRNA expressions in endometrial cells. **a**-**j** Endometrial cells obtained from young and aged cows were cultured and mRNA expressions which picked up in target molecules in canonical pathway were determined by quantitative RT-PCR. Data are expressed as the mean ± SEM (*n* = 5 cows in young and *n* = 7 cows in aged group). Significant differences were detected using the t-test; * *p* < 0.05

To confirm the data of RNA-seq analysis, mRNA expression of several key factors in each canonical pathways (already reported in previous studies [23–25]) were examined by quantitative RT-PCR (Fig. 1). Total RNA was prepared using ISOGEN (Nippon Gene Company, Limited, Toyama, Japan) according to the manufacturer’s instructions. After RNA extraction, cDNA production were performed a commercial kit (ReverTra Ace; Toyobo Co., Ltd., Osaka, Japan) and 0.5 μg RNA (approximately 1–3 μl/sample) was subjected to the cDNA synthesis. Real-time RT-PCR was performed using the CFX Connect™ Real Time PCR (Bio-Rad, Hercules, CA) and a commercial kit (Thunderbird SYBR qPCR Mix; Toyobo Co., Ltd.) to detect mRNA expressions of *CCL5, C1QA, MX1, MX2, STAT1, IRF1, IRF2, ISG15, CDK1, CCNB1* and *β-actin*. The following antisense and sense primers were used: *CCL5* (5′- CCTCCCCATATGCCTCG -3′ and 5′- TTGGCGCACACCTGG -3′ Accession No. NM_175827.2), *C1QA* (5′- CGTTGGACCGAATTCTGTCTC -3′ and 5′- TGCTGTTGAAGTCACAGAAGCC -3′ Accession No. NM_001014945.1), *MX1* (5′- GTCCCTGCTAACGTGGACAT -3′ and 5′- ACCAGGTTTCTCACCACGTC -3′ Accession No. NM_173940), *MX2* (5′- GCAGATCAAGGCACTCATCA -3′ and 5′- ACCAGGTCTGGTTTGGTCAG -3′ Accession No. NM_173941.2), *STAT1* (5′- CTCATTAGTTCTGGCACCAGC -3′ and 5′- CACACGAAGGTGATGAACATG -3′ Accession No. NM_001077900), *IRF1* (5′- GCTGGGACATCAACAAGGAT -3′ and 5′- CTGCTCTGGTCCTTCACCTC -3′ Accession No. NM_177432.2), *IRF2* (5′- AAACTGGGCCATCCATACAG -3′ and 5′- TTAGAAGGCCGCTCAGACAT -3′ Accession No. AJ490936.1), *ISG15* (5′- GGTATGATGCGAGCTGAAGCACTT -3′ and 5′- ACCTCCCTGCTGTCAAGGT -3′ Accession No. NM_174366), *CDK1* (5′- ATGGCTTGGATCTGCTCTCG -3′ and 5′- CATTAAAGTACGGATGATTCAGTGC -3′ Accession No. NM_174016), *CCNB1* (5′- TGGGTCGGCCTCTACCTTTGCACTTC -3′ and 5′- CGATGTGGCATACTTGTTCTTGATAGTCA -3′ Accession No. NM_001045872), and *β-actin* (5′- CCAAGGCCAACCGTGAGAAAAT -3′ and 5′- CCACATTCCGTGAGGATCTTCA -3′ Accession No. MN_173979.3). Real-time RT-PCR was performed in duplicate with a final reaction volume of 20 μl containing 10 μl SYBR Green, 7.8 μl distilled water, 0.1 μl 100 μM forward and reverse primers, and 2 μl of cDNA template. The amplification program consisted of a 5 min denaturation at 95 °C followed by 40 cycles of amplification (95 °C for 15 s, 60 °C for 30 s, and 72 °C for 20 s). Negative controls (RT samples without any RNA during cDNA synthesis) were subjected in each analysis. Expression levels of each target gene were normalized to corresponding *β-actin* threshold cycle (CT) values using the ΔΔ CT comparative method [26]. The specific melting point of the amplified product carried out as verification of the product identify. After real-time RT-PCR analysis, the PCR products were subjected to electrophoresis, and the target band was observed in the predicted size.

### Statistical analysis

Data are expressed as mean ± SEM. Differences between young and aged groups were identified using unpaired *t-*tests. Multiple comparisons were made using one-way analysis of variance (ANOVA) followed by Bonferroni’s multiple comparison tests. In cell culture experiment, a *p*-value of <0.05 was considered to be statistically significant.

## Results

### Predicted canonical pathways in endometrial cells obtained from young and aged cows

Our comparison of canonical pathways predicted by an ingenuity upstream regulator analysis implemented in an IPA based on differentially expressed genes in endometrial cells obtained from young and aged cows detected 355 canonical pathways (data not shown). Z-scores were used to determine whether canonical pathways had significantly more “activated” predictions or “inhibited” predictions. We selected the predicted canonical pathways with Z-scores greater than 2 or less than −2; 5 canonical pathways were extracted as shown in Table 1. Related molecules with each canonical pathway are also shown, respectively (Table 1). In addition, fold changes and RPKM values of these target molecules which predicted as each canonical pathways including Role of Pattern Recognition Receptors in Recognition of Bacteria and Viruses (Additional file 1: Table S1), Interferon signaling (Additional file 2: Table S2), and Cell Cycle: G2/M DNA Damage Checkpoint Regulation (Additional file 3: Table S3) are shown, respectively. To confirm the data of RNA-seq analysis, mRNA expression of several factors in each canonical pathways were examined by quantitative RT-PCR (Fig. 1). In predicted canonical pathway as Role of Pattern Recognition Receptors in Recognition of Bacteria and Viruses (Additional file 1: Table S1), although *C1QA* mRNA expression did not differ between endometrial cells obtained from young and aged cows (Fig. 1b), *CCL5* mRNA expression was significantly higher in endometrial cells obtained from aged compared with young cows (Fig. 1a). In predicted canonical pathway as Interferon signaling (Additional file 2: Table S2), *MX1* mRNA expression tended to be higher (Fig. 1c), and *MX2* mRNA expression was significantly higher in endometrial cells obtained from aged (RPKM value = 492) compared with young cows (RPKM vale = 84, data not shown). In addition, similar to the results of the RNA-seq analysis, *STAT1* and *ISG15* mRNA expression were significantly higher and *IRF1* mRNA expression tended to be higher in endometrial cells obtained from aged compared with young cows (Fig. 1e, f, and h). According to the RNA-seq analysis, the mRNA expression levels of *IRF2* were similar in endometrial cells obtained from young (RPKM value = 451) and aged cows (RPKM value = 547, relative fold changes aged/young: 1.21). We confirmed that the *IRF2* mRNA expression did not differ between young and aged cows (Fig. 1g). Finally, in predicted canonical pathway as Cell Cycle: G2/M DNA Damage Checkpoint Regulation (Additional file 3: Table S3), *CDK1* mRNA expression (Fig. 1i) was significantly lower levels and *CCNB1* mRNA expression (Fig. 1j) also tended to be lower in endometrial cells obtained from aged compared with young cows. These data suggested that although it did not completely match, we were able to confirm the results of the RNA-seq data by using of quantitative RT-PCR in the present study.

**Table 1:** Comparison of canonical pathways between bovine young and aged endometrial cells

Rank	Name of canonical pathways	zScore	*p*-value	Molecules
1	Role of Pattern Recognition Receptors in Recognition of Bacteria and Viruses	3.16	4.68E-04	IFIH1, IL1A, C5AR1, PIK3CG, DDX58, PIK3R6, C1QA, C1QC, CLEC6A, CCL5, C1QB, CSF2
2	Interferon Signaling	2.83	1.05E-05	IFIT1, IFIT3, MX1, PSMB8, STAT1, TAP1, IRF1, ISG15
3	Cell Cycle: G2/M DNA Damage Checkpoint Regulation	2.83	1.55E-05	CDC25B, CDC25C, CKS2, TOP2A, CCNB2, PLK1, AURKA, CDK1, CCNB1
4	Complement System	2.24	9.77E-04	CFD, ITGB2, C5AR1, C1QA, C1QC, C1QB
Rank	Name of canonical pathways	zScore	*p*-value	Molecules
1	Mitotic Roles of Polo-Like Kinase	−2.31	1.00E-08	KIF23, CDC25C, ESPL1, CDC20, PTTG1, PRC1, CCNB2, PLK1, CDK1, CCNB1, CDC25B, PLK4, PPP2R2B, KIF11

In addition, we identified the main signaling pathways associated with diseases and bio-functions. The top categories with an increase or decrease are shown in Additional file 4: Table S4. The predicted functions of the top category with an increase were involved with inflammatory responses (immune response of cells). The predicted functions of the top category with a decrease were related to Infection diseases (replication of virus). Related molecules for each category are shown, respectively. Interestingly, inflammatory signaling-related molecules (*CXCL10*, *MERTK*, and *TNFSF10*) and interferon signaling-related molecules (*ISG15*, *RSAD2*, and *MX2*) were clearly upregulated in endometrial cells obtained from aged compared with young cows. The above results suggest that inflammatory signaling, IFN signaling, and cell cycle/cell division are the main functions associated with age-dependent changes in bovine endometrial cells.

**Fig. 2:**
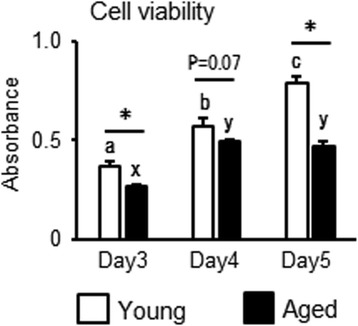
Age-dependent changes in endometrial cell viability. Endometrial cells obtained from young and aged cows were cultured and cell viability was determined using the WST1 assay. Data are expressed as the mean ± SEM (*n* = 5 cows in young and *n* = 7 cows in aged group). Significant differences were detected using ANOVA followed by Bonferroni’s multiple comparison test; * *p* < 0.05

We compared characteristic endometrial cell functions of young and aged bovine uteri in culture. On the basis of the above analysis, which predicted activation of Cell Cycle: G2/M DNA Damage Checkpoint Regulation and inhibition of Mitotic Roles of PLK, we hypothesized that aged endometrial cells accumulated DNA damage, resulting in cell cycle arrest and a cessation of cell division. Therefore, we examined the effect of aging on cell viability using WST1 cell proliferation reagent solution. Interestingly, although cell viability of endometrial cells obtained from young cows was significantly increased as day-dependent manner, and that of aged cows did not increased in later half despite having not reached confluence of cells (Fig. 2). In addition, despite seeding with the same number of endometrial cells, endometrial cells obtained from young cows exhibited significantly higher cell proliferation compared with aged cows (Fig. 2).

### Predicted upstream regulators in endometrial cells obtained from young and aged cows

Next, we identified upstream regulators associated with aging of bovine endometrial cells. We picked the three top-ranked activated or inhibited upstream regulators, as shown in Table 2 and other predicted upstream regulator are shown in Additional file 5: Table S5. This comparison predicted that interferon regulator factor (IRF) 7, tretinoin, and IFNG as activated upstream regulators in aged endometrial cells, whereas prostaglandin (PG) E2 receptor 2 (PTGER2), T-Box protein 2 (TBX2), and IL1 receptor antagonist (IL1RA) were predicted as inhibited upstream regulators in endometrial cells obtained from aged cows. Related mechanistic networks with each upstream regulator are shown, respectively (Table 2).

**Table 2:** Comparison of upstream regulator between bovine young and aged endometrial cells

Rank	Upstream regulator	*p*-value	Activation z-score	Prediction activation state	Mechanistic network
1	IRF7	3.28E-22	5.835	Activated by Aged	CREBBP, IFNA1/IFNA13, IFNA2, IFNB1, IRF1, IRF3, IRF7, ISGF3, IFNA, NFkB (complex), STAT1, STAT2, STAT3, STAT4
2	tretinoin	1.96E-35	5.543	Activated by Aged	CEBPA, ETS1, FOXO3, IFNG, IL-6, IRF1, IRF8, NFKB1, NFkB (complex), RELA, SP1, STAT1, STAT3, TGFB1, TGM2, TNF, TP53, tretinoin
3	IFNG	5.51E-36	5.496	Activated by Aged	CREBBP, IFNG, IL-10, IL-1B, IRF1, IRF7, IRF8, IRF9, NFKB1, NFKBIA, NFkB (complex), RELA, STAT1, STAT2, STAT3, TNF
Rank	Upstream regulator	*p*-value	Activation z-score	Prediction activation state	Mechanistic network
1	PTGER2	6.89E-34	−4.866	Inhibited by Aged	Ap1, CSF2, FOXO3, IL-6, IRF1, NFKBIA, NFkB (complex), PTGER2, Rb, SP1, STAT3, TP53
2	TBX2	2.17E-11	−4.111	Inhibited by Aged	CCND1, CDKN1A, E2F1, E2F4, E2f, HDAC1, HDAC2, RB1, Rb, SMAD7, TBX2, TP53
3	IL1RN	4.18E-15	−4.08	Inhibited by Aged	Ap1, IFNG, IFNL1, IL-1A, IL-1B, IL1RN, IRF1, IRF8, NFKB1, NFKBIA, NFkB (complex), RELA, STAT1, STAT2, STAT3, STAT4, TNF

### Predicted canonical pathways and upstream regulators after IFNT challenge in endometrial cells obtained from young and aged cells

To test our hypothesis that the effect of IFNT will change depending on the age of cattle, we comprehensively analyzed the influence of IFNT in bovine endometrial cells by aging using a next-generation sequencer. First, we selected the predicted canonical pathways with Z-scores greater than 2 or less than −2; the top 5 canonical pathways were extracted as shown in Table 3. This comparison predicted that Interferon signaling, Retinoic acid Mediated Apoptosis Signaling, Role of Pattern Recognition Receptors in Recognition of Bacteria and Viruses, Death Receptor Signaling, and UVA-induced MAPK signaling as activated canonical pathways both in endometrial cells obtained from young and aged cells after IFNT treatment. Although the order of rankings and target molecules were slightly different, the predicted activated canonical pathways after IFNT treatment completely matched between young and aged cells.

**Table 3:** Comparison of canonical pathways by IFNT treatment in bovine young and aged endometrial cells

Young	Name of canonical pathways	zScore	*p*-value	Molecules
1	Interferon Signaling	2.65	1.58E-13	IFITM3, IFIT1, IFIT3, MX1, IRF9, IFI6, IFITM2, ISG15
2	Retinoic acid Mediated Apoptosis Signaling	2.24	1.29E-06	PARP10, TNFSF10, PARP12, PARP9, PARP14
3	Role of Pattern Recognition Receptors in Recognition of Bacteria and Viruses	2.00	2.45E-06	IFIH1, IL-1A, IRF7, OAS2, DDX58, EIF2AK2
4	Death Receptor Signaling	2.24	9.77E-06	PARP10, TNFSF10, PARP12, PARP9, PARP14
5	UVA-induced MAPK signaling	2.00	1.62E-04	PARP10, PARP12, PARP9, PARP14
Aged	Name of canonical pathways	zScore	*p*-value	Molecules
1	Interferon Signaling	2.45	1.66E-09	IFIT1, IFIT3, MX1, IFI6, IFITM2, ISG15
2	Role of Pattern Recognition Receptors in Recognition of Bacteria and Viruses	2.00	5.37E-05	IFIH1, IRF7, OAS2, DDX58, EIF2AK2
3	Retinoic acid Mediated Apoptosis Signaling	2.00	5.50E-05	PARP10, PARP12, PARP9, PARP14
4	UVA-induced MAPK signaling	2.00	1.99E-04	PARP10, PARP12, PARP9, PARP14
5	Death Receptor Signaling	2.00	2.24E-04	PARP10, PARP12, PARP9, PARP14

Next, we identified upstream regulators after IFNT treatment depending on the age of bovine endometrial cells. We selected predicted upstream regulators with activation Z-scores greater than 2 or less than −2, and *p*-value <0.00001 was used for data in this analysis. We picked the higher-ranked activated or inhibited upstream regulators as shown in Table 4. This comparison predicted that IRF7, eukaryotic translation initiation factor 2-alpha kinase 2 (EIF2AK2), and DExD/H-box helicase 58 (DDX58, also named RIG-I) as activated upstream regulators by IFNT stimulus, whereas ubiquitin specific peptidase 18 (USP18) was predicted as an inhibited upstream regulator by IFNT stimulus both in endometrial cells obtained from young and aged cows.

**Table 4:** Comparison of upstream regulator by IFNT treatment in bovine young and aged endometrial cells

Young	Upstream regulator	*p*-value	Activation z-score	Prediction activation state	Aged	Upstream regulator	*p*-value	Activation z-score	Prediction activation state
1	IRF7	2.44E-48	5.350	Activated by IFNT	1	IRF7	8.25E-54	5.603	Activated by IFNT
2	EIF2AK2	1.40E-19	2.893	Activated by IFNT	2	EIF2AK2	2.09E-17	3.426	Activated by IFNT
3	DDX58	2.82E-20	2.635	Activated by IFNT	3	DDX58	4.47E-22	2.818	Activated by IFNT
Young	Upstream regulator	*p*-value	Activation z-score	Prediction activation state	Aged	Upstream regulator	*p*-value	Activation z-score	Prediction activation state
1	USP18	2.82E-14	−2.592	Inhibited by Aged	1	USP18	1.51E-09	−2.213	Inhibited by Aged

### Comparison of upregulated molecules after IFNT challenge in endometrial cells obtained from young and aged cells

Finally, we investigated the integrated effects of IFNT depending on the age of bovine endometrial cells based on the transcription levels of individual genes. According to the RNA-seq analysis, relative fold changes were calculated using RPKM value (fold changes IFNT/control). The top 10 activated genes (higher fold changes) in endometrial cells obtained from young cows are listed in Table 5 (left side). All activated genes are known as IFN-inducible genes (ISGs) [1]. Next, we listed these 10 genes in endometrial cells obtained from aged cows (Table 5, right side) and compared the fold changes after IFNT treatment between young and aged cells. Although all genes in aged cells were also activated by IFNT treatment, the rate of increase (fold changes) by IFNT stimulus was obviously lower in the endometrial cells obtained from aged compared to young cows.

**Table 5:** Upregulated molecules by IFNT treatment in bovine young endometorial cells and comparison with aged endometrial cells

Rank	Molecules	Young control RPKM	Young IFNT RPKM	Fold change	Aged Control RPKM	Aged IFNT RPKM	Fold change
1	OAS2	0.17	2.28	13.46	0.87	1.80	2.08
2	MX2	0.86	9.01	10.54	5.40	17.01	3.15
3	OAS1Z	4.37	38.87	8.90	15.04	38.35	2.55
4	OAS1Y	12.78	99.88	7.81	42.87	101.79	2.37
5	ISG15	16.55	129.29	7.81	78.99	172.74	2.19
6	ZPB1	1.57	10.77	6.86	7.06	13.13	1.86
7	USP18	2.57	16.22	6.31	7.30	17.10	2.34
8	MX1	20.89	119.31	5.71	70.55	162.91	2.31
9	RSAD2	2.26	12.91	5.70	15.34	31.81	2.07
10	IFI44	0.41	2.31	5.59	1.53	4.47	2.93

**Fig. 3:**
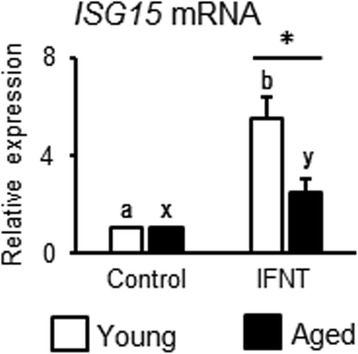
Age-dependent changes in IFNT response in endometrial cells. Endometrial cells obtained from young and aged cows were cultured. IFNT (1 ng/mL) were treated for 24 h and mRNA expression of *ISG15* was determined by quantitative RT-PCR. Data are expressed as the mean ± SEM (*n* = 5 cows in young and *n* = 7 cows in aged group). Significant differences were detected using ANOVA followed by Bonferroni’s multiple comparison test; * *p* < 0.05

IFN-stimulated gene 15 (ISG15) is a well-known ISG and IFNT clearly stimulates *ISG15* mRNA expression in bovine endometrial cells [27]. The changes in *ISG15* transcription detected by RNA-seq analysis were confirmed, *ISG15* mRNA expression significantly stimulated by IFNT treatment both in endometrial cells obtained from young and aged cows (Fig. 3). In addition, the increased levels of *ISG15* mRNA expression were higher in endometrial cells obtained from young compared with aged cows after IFNT treatment (Fig. 3).

Interestingly, 4 molecules activated by IFNT that fluctuated only in endometrial cells obtained from aged cows were detected, as shown in Additional file 6: Table S6. The treatment with IFNT increased tumor protein translationally controlled 1 (*TPT1*) and ribosomal protein L17 (*RPL17*) in aged but decreased these genes in young cows. On the other hand, chemokine (C-X-C motif) ligand *(CXCL) 9* and *CXCL10* were increased (fold change; two or more) by IFNT treatment in endometrial cells obtained from aged but not young cows.

## Discussion

There is little information regarding age-dependent changes in uterine functions. We defined cows aged more than 150 months as old, and cows aged between 28 and 68 months as young. Then, we performed next generation sequencing and demonstrated that mRNA expressions in bovine endometrial cells are age-dependently changes, especially pro-inflammatory signaling-, interferon signaling-, and cell cycle/cell division-related components.

In the present study, the top ranked canonical pathway (Table 1) and diseases and bio functions (Additional file 4: Table S4) were “Role of Pattern Recognition Receptors in Recognition of Bacteria and Viruses” and “Inflammatory response”, respectively, which were predicted to be activated in endometrial cells obtained from aged cows. These findings suggest that inflammatory signaling spontaneously increases with age in cow endometrial cells. Similarly, Elmes et al. [16] reported that maternal age affected the upregulation of genes related to immune and inflammatory responses in rat uteri. IL1A, as a candidate molecule within the predicted canonical pathways, is one of the essential pro-inflammatory cytokines. Age-related increases in IL1A have also been reported in endometrial stromal fibroblasts, endothelial cells, and ovaries [28, 29]. Uri-Belapolsky et al. [30] reported that *Il1α* deficient mice exhibited a higher pregnancy rate (81%) with higher litter sizes in aged mice compared with wild-type aged mice (pregnancy rate: 53%). In addition, Gardner et al. [31] demonstrated that senescent vascular smooth muscle cells secrete multiple inflammatory cytokines in an IL1A-dependent manner. Therefore, it has been suggested that an age-dependent increase in inflammatory responses, including IL1A, are associated with endometrial cellular senescence, resulting in reproductive dysfunction.

Recently, it has been reported that IFN activity and its signaling are elevated with aging, contributing to the aging-related diseases in humans and mice [32, 33]. To support these results, we showed that Interferon Signaling as a canonical pathway and IRF7 and IFNG as upstream regulators were activated in aging endometrial cells compared with young cells. Indeed, Yu et al. [34] reported that IFNB activates the p53-p21 axis, promoting cell senescence in vitro. On the other hand, type I IFNs bind to common receptors such as IFNA receptor 1 (IFNAR1) and 2 (IFNAR2) [35]. The Janus kinases (JAKs) –signal transducer and activator of transcription (STAT) pathways are major regulators of the transcription of ISGs [35]. Interestingly, JAK-STAT signaling and ISG15 expression were also spontaneously enhanced dependent on aging of epidermal stem cells, skeletal muscle, and skin in humans and mice [32, 33, 36]. In addition, a shorter telomere (as a marker of senescence) induced ISG15 expression together with inflammation and cellular senescence (β-galactosidase activity), whereas the induction of a longer telomere resulted in a lower grade ISG15 expression and cellular senescence, suggesting that upregulation of ISG15 with telomere shortening may contribute to chronic inflammatory states in human aging [36]. Therefore, we suggest that an age-dependent increase of IFN-JAK-STAT-ISGs systems are associated with the promotion of uterine aging in cows.

Mitotic checkpoint genes are believed to be prime targets for deregulation in human infertility [37]. Actually, Jin et al. [38] demonstrated that Cdc20, the activating subunit of the anaphase-promoting complex/cyclosome, was critical for meiosis and fertility in mice. They showed that female mice with low amounts of Cdc20 almost exclusively produced aneuploidy embryos, resulting in failure to thrive and death early in development, and suggested the possibility that Cdc20 insufficiency may be a cause of infertility [38]. In the present study, the comparison of canonical pathways represented the activation of “Cell Cycle: G2/M DNA Damage Checkpoint Regulation” and the inhibition of “Mitotic Role of PLK” in aged endometrial cells, hypothesizing that the accumulation of DNA damage with aging of endometrial cells and the stopping of cell proliferation due to cell cycle arrest. To support this hypothesis, we observed the lower proliferative levels of endometrial cells obtained from aged compared with young cows. Also, we observed the different speed to confluence in endometrial cells obtained from between young and aged cows (observation by microscopy). In addition, mammalian tissues from aged individuals retain unrepaired DNA damage (DNA double-strand breaks) associated with DNA damage response [39], and an inflammatory situation induces fragmented DNA in the preimplantation-stage of embryos and uterine cells, causing poor embryonic development and improper preparation of the uterine microenvironment for pregnancy [40]. Furthermore, tissues with accumulating DNA damage produce endogenous IFNs and stimulate IFN signaling, promoting IFN-induced senescence and amplifying DNA damage responses [34]. These above findings with our present data suggest that bovine endometrial cell aging is associated with an imbalance in the cell cycle due to the accumulation of DNA damage and hyper activation of IFN signaling.

In contrast to data showing the activating Interferon Signaling as upstream regulators, we showed that PTGER2 as an upstream regulator was inhibited in endometrial cells obtained from aged cows compared with young cows. PTGER2 is one of the specific receptors for PGE2. The importance of the PGE2-PTGER2 axis in pregnancy has been well demonstrated for various phenomena and it has multiple roles in ovulation, fertilization via expansion of oocytes from cumulus cells, embryo development, maternal recognition, and implantation [41–45]. Therefore, we suggest that downregulation of PTGER2 signaling with aging may lead to infertility or a declining pregnancy rate. Indeed, PGE2 concentrations increased significantly in human endometrial fluid during the window of implantation, and PGE2 levels were markedly lower in patients who did not achieve pregnancy [46]. Interestingly, Peloffo et al. [47] reported that the chronic administration of a selective PTGER2 antagonist resulted in a significant contraceptive effect without adverse side effects in female macaques. These findings suggest that endometrial cells obtained from aged cows have the potential to reduce embryo development via lower PGE2-PTGER2 signaling. In future research, we plan to investigate age-dependent interactions between the early embryo and endometrial cells.

To address the issue from a different angle, we asked that signal transduction by IFNT changed with aging in endometrial cells in cows. To test our hypothesis, we comprehensively analyzed the influence of IFNT on bovine endometrial cells depending on aging using a next-generation sequencer. Contrary to our expectation, the analysis of canonical pathways and upstream regulators revealed that there was no difference in the effect of IFNT depending on the age of bovine endometrial cells (Table 4). As results in Table 4, it has been reported that type I IFNs including IFNT regulate these three activated upstream regulators (IRF7, EIF2AK2, and DDX58) [1, 48], whereas USP18 acts as an inhibitor of IFN signal transduction pathways [49]. Similar to the data of predicted canonical pathways, the predicted upstream regulators after IFNT treatment also completely matched between endometrial cells obtained from young and aged cows. These findings indicated that the influence of IFNT is almost the same between endometrial cells obtained from young and aged cows. Gierek et al. [50] reported that a low IFNT dose (0.01 and 0.1 ng/mL) had a less inhibitory effect in the proliferation of lymphocytes in cows (multiparous), whereas the same dose IFNT clearly inhibited lymphocyte proliferation in heifers (15–16 months of age), indicating that lymphocytes from young heifers were more susceptible to IFNT treatment. Similarly, we found that the stimulatory effect of IFNT was obviously higher in endometrial cells obtained from young cows than that in aged cows. Possible reasons for the different responsiveness to IFNT could be: (1) IFNT responsiveness may change according to the situation such as advancing age or pregnancy experience, and (2) basic levels of interferon signaling and ISGs are high as endometrial cells advance in age, resulting in the relatively low responsiveness to IFNT treatment.

A limitation of the present study was the use of in vitro bovine endometrial cell models: the use of in vitro models to study in vivo situations does not reflect the in vivo condition perfectly. Therefore, in vivo investigations about the effect of aging in bovine endometrium functions are required to clarify these issues. In addition, although endometrial cell function at days 10–15 of the estrous cycle changed with aging, we did not investigate the aging effect in endometrial cell function using other estrous cycle periods or pregnancy state. Moreover, because we wanted to verify the comprehensive change with age in the bovine endometrium, we did not distinguish between epithelial and stromal cells in uterine tissues when we isolated cells with reference the past investigation [21]. However, because these cells exert different functions in an in vivo situation, the effect of aging may be changed depending on cell types. Thus, further investigations are required to clarify these issues.

## Conclusion

We demonstrated that comprehensive gene characteristics of bovine endometrial cells differed depending on their age. Endometrial cells obtained from aged cows exhibited spontaneously higher levels of inflammatory signaling and IFN signaling, and dysfunction of cell division was associating with chronic inflammation, activation of IFN signaling, and accumulation of DNA damage. A high basal level of IFN-related mRNA expressions in endometrial cells of aged cows may lead to lower susceptible for IFNT compared to younger ones, which is suggested as “inflammaing” in bovine endometrial cells. Further investigations are needed to elucidate age-dependent functional changes in the uterus, to understand its physiological roles during pregnancy, and the pathophysiology of complications in pregnancy related to the uterus.

## Additional files

Additional file 1: Canonical pathways-related molecules: Role of Pattern Recognition Receptors in Recognition of Bacteria and Viruses. (DOCX 15 kb)

Additional file 2: Canonical pathways-related molecules: Interferon Signaling. (DOCX 15 kb)

Additional file 3: Canonical pathways-related molecules: Cell Cycle: G2/M DNA Damage Checkpoint Regulation. (DOCX 15 kb)

Additional file 4: Comparison of Diseases and Bio Functions between bovine young and aged endometrial cells. (DOCX 15 kb)

Additional file 5: Comparison of upstream regulator between bovine young and aged endometrial cells. (DOCX 17 kb)

Additional file 6: Upregulated molecules by IFNT treatment in bovine aged endometrial cells and comparison with young endometrial cells. (DOCX 16 kb)

## Abbreviations

CDC20: Cell-division cycle protein 20
CSF2: Colony-stimulating factor 2
CXCL: C-X-C motif chemokine
DDX58: DExD/H-box helicase 58
EIF2AK2: Eukaryotic translation initiation factor 2-alpha kinase 2
IFNG: Interferon gamma, IFNAR, interferon A receptor
IFNT: Interferon tau
IL1A: Interleukin-1alpha
IL1RA: IL1 receptor antagonist
IRF: Interferon regulator factor
ISG: Interferon stimulated gene
JAK: Janus kinase
MERTK: Proto-oncogene tyrosine kinase MER
MX: Myxovirus resistance
PGE2: Prostaglandin E2
PLK: Polo-like kinase
PTGER2: PGE receptor 2
RPL17: Ribosomal protein L17
RSAD2: Radical S-adenosyl methionine domain containing 2
STAT: Signal transducer and activation of transcription
TBX2: T-Box protein 2
TNFSF10: Tumor necrosis factor ligand superfamily member 10
TPT1: Tumor protein translationally controlled 1
USP18: Ubiquitin specific peptidase 18

## References

[1] Yang L, Zhang LY, Qiao HY, Liu N, Wang YX, Li SJ (2014). Maternal immune regulation by conceptus during early pregnancy in the bovine. Asian J Anim Vet Adv.

[2] Imakawa K, Anthony RV, Kazemi M, Marotti KR, Polites HG, Roberts RM (1987). Interferon-like sequence of ovine trophoblast protein secreted by embryonic trophectoderm. Nature.

[3] Bazer FW, Roberts RM (1983). Biochemical aspects of conceptus--endometrial interactions. J Exp Zool.

[4] Ashworth CJ, Bazer FW (1989). Changes in ovine conceptus and endometrial function following asynchronous embryo transfer or administration of progesterone. Biol Reprod.

[5] Roberts RM, Chen Y, Ezashi T, Walker AM (2008). Interferons and the maternal-conceptus dialog in mammals. Semin Cell Dev Biol.

[6] Jabbour HN, Sales KJ, Catalano RD, Norman JE (2009). Inflammatory pathways in female reproductive health and disease. Reproduction.

[7] Carneiro LC, Cronin JG, Sheldon IM (2016). Mechanisms linking bacterial infections of the bovine endometrium to disease and infertility. Reprod Biol.

[8] Lim MA, Lee J, Park JS, Jhun JY, Moon YM, Cho ML, Kim HY (2014). Increased Th17 differentiation in aged mice is significantly associated with high IL-1beta level and low IL-2 expression. Exp Gerontol.

[9] Schmitt V, Rink L, Uciechowski P (2013). The Th17/Treg balance is disturbed during aging. Exp Gerontol.

[10] Goto M (2008). Inflammaging (inflammation + aging): a driving force for human aging based on an evolutionarily antagonistic pleiotropy theory?. Biosci Trends.

[11] Jagger A, Shimojima Y, Goronzy JJ, Weyand CM (2014). Regulatory T cells and the immune aging process: a mini-review. Gerontology.

[12] Alvarez-Rodriguez L, Lopez-Hoyos M, Munoz-Cacho P, Martinez-Taboada VM (2012). Aging is associated with circulating cytokine dysregulation. Cell Immunol.

[13] Malhi PS, Adams GP, Mapletoft RJ, Singh J (2007). Oocyte developmental competence in a bovine model of reproductive aging. Reproduction.

[14] Nelson SM, Telfer EE, Anderson RA (2013). The ageing ovary and uterus: new biological insights. Hum Reprod Update.

[15] Tanaka H, Ohtsu A, Shiratsuki S, Kawahara-Miki R, Iwata H, Kuwayama T, Shirasuna K (2016). Age-dependent changes in inflammation and extracellular matrix in bovine oviduct epithelial cells during the post-ovulatory phase. Mol Reprod Dev.

[16] Elmes M, Szyszka A, Pauliat C, Clifford B, Daniel Z, Cheng Z, Wathes C, McMullen S. Maternal age effects on myometrial expression of contractile proteins, uterine gene expression, and contractile activity during labor in the rat. Physiol Rep. 2015;3:e12305. 10.14814/phy2.12305 PMC4425948 25876907

[17] Hirota Y, Daikoku T, Tranguch S, Xie H, Bradshaw HB, Dey SK (2010). Uterine-specific p53 deficiency confers premature uterine senescence and promotes preterm birth in mice. J Clin Invest.

[18] Miyamoto A, Schams D (1991). Oxytocin stimulates progesterone release from microdialyzed bovine corpus luteum in vitro. Biol Reprod.

[19] Kowsar R, Jiemtaweeboon S, Shirasuna K, Shimizu T, Sasaki M, Kitamura N, Miyamoto A (2014). Accumulation of eosinophils in the infundibulum of the bovine oviduct just after ovulation. J Vet Med Sci.

[20] Ireland JJ, Murphee RL, Coulson PB (1980). Accuracy of predicting stages of bovine estrous cycle by gross appearance of the corpus luteum. J Dairy Sci.

[21] Boruszewska D, Kowalczyk-Zieba I, Sinderewicz E, Grycmacher K, Staszkiewicz J, Woclawek-Potocka I (2017). The effect of lysophosphatidic acid together with interferon tau on the global transcriptomic profile in bovine endometrial cells. Theriogenology.

[22] Shirasuna K, Matsumoto H, Kobayashi E, Nitta A, Haneda S, Matsui M, Kawashima C, Kida K, Shimizu T, Miyamoto A (2012). Upregulation of interferon-stimulated genes and interleukin-10 in peripheral blood immune cells during early pregnancy in dairy cows. J Reprod Dev.

[23] Bauersachs S, Ulbrich SE, Gross K, Schmidt SE, Meyer HH, Wenigerkind H, Vermehren M, Sinowatz F, Blum H, Wolf E (2006). Embryo-induced transcriptome changes in bovine endometrium reveal species-specific and common molecular markers of uterine receptivity. Reproduction.

[24] Kim MS, Min KS, Imakawa K (2013). Regulation of interferon-stimulated gene (ISG)12, ISG15, and MX1 and MX2 by Conceptus Interferons (IFNTs) in bovine uterine epithelial cells. Asian-Australas J Anim Sci.

[25] Thelie A, Papillier P, Pennetier S, Perreau C, Traverso JM, Uzbekova S, Mermillod P, Joly C, Humblot P, Dalbies-Tran R (2007). Differential regulation of abundance and deadenylation of maternal transcripts during bovine oocyte maturation in vitro and in vivo. BMC Dev Biol.

[26] Livak KJ, Schmittgen TD (2001). Analysis of relative gene expression data using real-time quantitative PCR and the 2(−Delta Delta C(T)) method. Methods.

[27] Ott TL, Yin J, Wiley AA, Kim HT, Gerami-Naini B, Spencer TE, Bartol FF, Burghardt RC, Bazer FW (1998). Effects of the estrous cycle and early pregnancy on uterine expression of Mx protein in sheep (Ovis Aries). Biol Reprod.

[28] Maier JA, Voulalas P, Roeder D, Maciag T (1990). Extension of the life-span of human endothelial cells by an interleukin-1 alpha antisense oligomer. Science.

[29] Kumar S, Millis AJ, Baglioni C (1992). Expression of interleukin 1-inducible genes and production of interleukin 1 by aging human fibroblasts. Proc Natl Acad Sci U S A.

[30] Uri-Belapolsky S, Shaish A, Eliyahu E, Grossman H, Levi M, Chuderland D, Ninio-Many L, Hasky N, Shashar D, Almog T, Kandel-Kfir M, Harats D (2014). Interleukin-1 deficiency prolongs ovarian lifespan in mice. Proc Natl Acad Sci U S A.

[31] Gardner SE, Humphry M, Bennett MR, Clarke MC (2015). Senescent vascular smooth muscle cells drive inflammation through an interleukin-1alpha-dependent senescence-associated Secretory phenotype. Arterioscler Thromb Vasc Biol.

[32] Price FD, von Maltzahn J, Bentzinger CF, Dumont NA, Yin H, Chang NC, Wilson DH, Frenette J, Rudnicki MA (2014). Inhibition of JAK-STAT signaling stimulates adult satellite cell function. Nat Med.

[33] Doles J, Storer M, Cozzuto L, Roma G, Keyes WM (2012). Age-associated inflammation inhibits epidermal stem cell function. Genes Dev.

[34] Yu Q, Katlinskaya YV, Carbone CJ, Zhao B, Katlinski KV, Zheng H, Guha M, Li N, Chen Q, Yang T, Lengner CJ, Greenberg RA (2015). DNA-damage-induced type I interferon promotes senescence and inhibits stem cell function. Cell Rep.

[35] Darnell JE, Kerr IM, Stark GR (1994). Jak-STAT pathways and transcriptional activation in response to IFNs and other extracellular signaling proteins. Science.

[36] Lou Z, Wei J, Riethman H, Baur JA, Voglauer R, Shay JW, Wright WE (2009). Telomere length regulates ISG15 expression in human cells. Aging (Albany NY).

[37] Vogt E, Kirsch-Volders M, Parry J, Eichenlaub-Ritter U (2008). Spindle formation, chromosome segregation and the spindle checkpoint in mammalian oocytes and susceptibility to meiotic error. Mutat Res.

[38] Jin F, Hamada M, Malureanu L, Jeganathan KB, Zhou W, Morbeck DE, van Deursen JM (2010). Cdc20 is critical for meiosis I and fertility of female mice. PLoS Genet.

[39] Galbiati A, Beausejour C, d'Adda di Fagagna F (2017). A novel single-cell method provides direct evidence of persistent DNA damage in senescent cells and aged mammalian tissues. Aging Cell.

[40] Jaiswal YK, Jaiswal MK, Agrawal V, Chaturvedi MM (2009). Bacterial endotoxin (LPS)-induced DNA damage in preimplanting embryonic and uterine cells inhibits implantation. Fertil Steril.

[41] Kennedy CR, Zhang Y, Brandon S, Guan Y, Coffee K, Funk CD, Magnuson MA, Oates JA, Breyer MD, Breyer RM (1999). Salt-sensitive hypertension and reduced fertility in mice lacking the prostaglandin EP2 receptor. Nat Med.

[42] Tilley SL, Audoly LP, Hicks EH, Kim HS, Flannery PJ, Coffman TM, Koller BH (1999). Reproductive failure and reduced blood pressure in mice lacking the EP2 prostaglandin E2 receptor. J Clin Invest.

[43] Hizaki H, Segi E, Sugimoto Y, Hirose M, Saji T, Ushikubi F, Matsuoka T, Noda Y, Tanaka T, Yoshida N, Narumiya S, Ichikawa A (1999). Abortive expansion of the cumulus and impaired fertility in mice lacking the prostaglandin E receptor subtype EP(2). Proc Natl Acad Sci U S A.

[44] Arosh JA, Banu SK, Chapdelaine P, Emond V, Kim JJ, MacLaren LA, Fortier MA (2003). Molecular cloning and characterization of bovine prostaglandin E2 receptors EP2 and EP4: expression and regulation in endometrium and myometrium during the estrous cycle and early pregnancy. Endocrinology.

[45] Arosh JA, Banu SK, Kimmins S, Chapdelaine P, Maclaren LA, Fortier MA (2004). Effect of interferon-tau on prostaglandin biosynthesis, transport, and signaling at the time of maternal recognition of pregnancy in cattle: evidence of polycrine actions of prostaglandin E2. Endocrinology.

[46] Vilella F, Ramirez L, Berlanga O, Martinez S, Alama P, Meseguer M, Pellicer A, Simon C (2013). PGE2 and PGF2alpha concentrations in human endometrial fluid as biomarkers for embryonic implantation. J Clin Endocrinol Metab.

[47] Peluffo MC, Stanley J, Braeuer N, Rotgeri A, Fritzemeier KH, Fuhrmann U, Buchmann B, Adevai T, Murphy MJ, Zelinski MB, Lindenthal B, Hennebold JD (2014). A prostaglandin E2 receptor antagonist prevents pregnancies during a preclinical contraceptive trial with female macaques. Hum Reprod.

[48] Song G, Fleming JA, Kim J, Spencer TE, Bazer FW (2011). Pregnancy and interferon tau regulate DDX58 and PLSCR1 in the ovine uterus during the peri-implantation period. Reproduction.

[49] Malakhova OA, Kim KI, Luo JK, Zou W, Kumar KG, Fuchs SY, Shuai K, Zhang DE (2006). UBP43 is a novel regulator of interferon signaling independent of its ISG15 isopeptidase activity. EMBO J.

[50] Gierek D, Baczynska D, Ugorski M, Bazer F, Kurpisz M, Bednarski T, Gorczykowski M, Chelmonska-Soyta A (2006). Differential effect of IFN-tau on proliferation and distribution of lymphocyte subsets in one-way mixed lymphocyte reaction in cows and heifers. J Reprod Immunol.

