# Supplementary material for: Age-associated mRNA expression changes in bovine endometrial cells in vitro
Source: Reprod Biol Endocrinol. 2017 Aug 14;15:63. doi: 10.1186/s12958-017-0284-z (PMC5556672; doi:10.1186/s12958-017-0284-z)
Supplement: Supplementary file 1 — Canonical pathways-related molecules: Role of Pattern Recognition Receptors in Recognition of Bacteria and Viruses. (DOCX 15 kb) [file 12958_2017_284_MOESM1_ESM.docx]

| Additional file 1: Table S1. Canonical patyways-related molecules: Role of Pattern Recognition Receptors in Recognition of Bacteria and Viruses | | | |
| --- | --- | --- | --- |
|  |  |  |  |
| Molecules | Exp fold changes Aged/Young | Young RPKM value | Aged RPKM value |
| IFIH1 | 2.80 | 365 | 1021 |
| IL1A | -2.78 | 842 | 303 |
| C5AR1 | 2.56 | 45 | 115 |
| PIK3CG | 2.39 | 180 | 431 |
| DDX58 | 2.32 | 316 | 732 |
| PIK3R6 | 2.43 | 93 | 226 |
| **C1QA** | 3.29 | 51 | 168 |
| C1QC | 3.35 | 37 | 124 |
| CLEC6A | 5.13 | 8 | 41 |
| **CCL5** | 3.39 | 85 | 288 |
| C1QB | 4.51 | 39 | 176 |
| CSF2 | -2.41 | 198 | 82 |
