# Supplementary material for: Age-associated mRNA expression changes in bovine endometrial cells in vitro
Source: Reprod Biol Endocrinol. 2017 Aug 14;15:63. doi: 10.1186/s12958-017-0284-z (PMC5556672; doi:10.1186/s12958-017-0284-z)
Supplement: Supplementary file 2 — Canonical pathways-related molecules: Interferon Signaling. (DOCX 15 kb) [file 12958_2017_284_MOESM2_ESM.docx]

| Additional file 2: Table S2. Canonical pathways-related molecules: Interferon Signaling | | | |
| --- | --- | --- | --- |
|  |  |  |  |
| Molecules | Exp fold changes Aged/Young | Young RPKM value | Aged RPKM value |
| IFIT1 | 2.12 | 661 | 1402 |
| IFIT3 | 4.26 | 35 | 149 |
| **MX1** | 3.13 | 1665 | 5214 |
| PSMB8 | 2.22 | 1279 | 2835 |
| **STAT1** | 1.97 | 4441 | 8732 |
| TAP1 | 3.14 | 1558 | 4899 |
| **IRF1** | 2.19 | 2443 | 5344 |
| **ISG15** | 4.42 | 330 | 1460 |
