# Supplementary material for: Age-associated mRNA expression changes in bovine endometrial cells in vitro
Source: Reprod Biol Endocrinol. 2017 Aug 14;15:63. doi: 10.1186/s12958-017-0284-z (PMC5556672; doi:10.1186/s12958-017-0284-z)
Supplement: Supplementary file 3 — Canonical pathways-related molecules: Cell Cycle: G2/M DNA Damage Checkpoint Regulation. (DOCX 15 kb) [file 12958_2017_284_MOESM3_ESM.docx]

| Additional file 3: Table S3. Canonical pathways-related molecules:Cell Cycle: G2/M DNA Damage Checkpoint Regulation | | | |
| --- | --- | --- | --- |
|  |  |  |  |
| Molecules | Exp fold changes Aged/Young | Young RPKM value | Aged RPKM value |
| CDC25B | -3.36 | 932 | 277 |
| CDC25C | -2.33 | 193 | 83 |
| CKS2 | -2.74 | 917 | 335 |
| TOP2A | -3.33 | 3104 | 933 |
| CCNB2 | -3.33 | 509 | 153 |
| PLK1 | -2.73 | 879 | 322 |
| **CDK1** | -3.23 | 778 | 241 |
| **CCNB1** | -3.30 | 1351 | 410 |
