# Supplementary material for: Age-associated mRNA expression changes in bovine endometrial cells in vitro
Source: Reprod Biol Endocrinol. 2017 Aug 14;15:63. doi: 10.1186/s12958-017-0284-z (PMC5556672; doi:10.1186/s12958-017-0284-z)
Supplement: Supplementary file 4 — Comparison of Diseases and Bio Functions between bovine young and aged endometrial cells. (DOCX 15 kb) [file 12958_2017_284_MOESM4_ESM.docx]

| Additional file 4: Table S4. Comparison of Diseases and Bio Functions between bovine young and aged endometrial cells | | | | | |
| --- | --- | --- | --- | --- | --- |
|  |  |  |  |  |  |
| Categories | Diseases or Functions | Activation zScore | Prediction activation state | Top four upregulated molecules | Top four downregulated molecules |
| Inflammatory response | immune response of cells | 3.333 | Activated by Aged | CXCL10, MERTK, ISG15, TNFSF10 | DMBT1, SCN5A, TOP2A, PLP1 |
| Infection diseases | replication of virus | 3.175 | Inhibited by Aged | RSAD2, CXCL10, MX2, ISG15 | KIF11, CCNA2, PTPRN, CIT |
