# Supplementary material for: Age-associated mRNA expression changes in bovine endometrial cells in vitro
Source: Reprod Biol Endocrinol. 2017 Aug 14;15:63. doi: 10.1186/s12958-017-0284-z (PMC5556672; doi:10.1186/s12958-017-0284-z)
Supplement: Supplementary file 5 — Comparison of upstream regulator between bovine young and aged endometrial cells. (DOCX 17 kb) [file 12958_2017_284_MOESM5_ESM.docx]

| Additional file 5: Table S5. Comparison of upstream regulator between bovine young and aged endometrial cells | | | | | |
| --- | --- | --- | --- | --- | --- |
|  |  |  |  |  |  |
| Rank | Upstream regulator | p-value | Activation z-score | Prediction activation state | Mechanistic Network |
| 4 | IFNA | 3.26E-14 | 5.038 | Activated by Aged | CREBBP, IFNB1, IFNG, IRF1, IRF3, IRF7, IRF8, IRF9, Interferon alpha, NFkB (complex), RELA, STAT1, STAT2, STAT3, STAT4 |
| 5 | IFNA2 | 2.03E-19 | 4.985 | Activated by Aged | CEBPA, CREBBP, IFNA2, IRF1, IRF7, IRF8, IRF9, MAPK1, NCOA2, NFKB1, NFkB (complex), RELA, RNA polymerase II, STAT1, STAT2, STAT3, STAT4 |
| 6 | NUPR1 | 4.34E-09 | 4.964 | Activated by Aged | CCND1, CDK4, NUPR1 |
| 7 | poly rI:rC-RNA | 3.39E-20 | 4.897 | Activated by Aged | CREBBP, IFN Beta, IFNG, IRF1, IRF7, IRF8, IRF9, Interferon alpha, NFKB1, NFKBIA, NFkB (complex), RELA, STAT1, STAT2, STAT3, poly rI:rC-RNA |
| 8 | STAT1 | 8.78E-27 | 4.516 | Activated by Aged | CEBPA, FOXO3, IFNG, IL1B, IRF1, IRF8, IRF9, Ifn, NFKB1, NFkB (complex), RELA, RNA polymerase II, STAT1, STAT2, STAT3, STAT4 |
| 9 | TGM2 | 2.99E-13 | 4.512 | Activated by Aged | Ap1, ERK1/2, NFKB1, NFKBIA, NFkB (complex), RELA, STAT1, STAT3, TGM2 |
| 10 | IFNAR | 9.20E-17 | 4.357 | Activated by Aged | CREBBP, IFNB1, IFNG, IL1B, IRF1, IRF3, IRF7, IRF8, IRF9, Ifnar, NFKB1, NFkB (complex), RELA, STAT1, STAT2, STAT3, poly rI:rC-RNA |
| 11 | TP53 | 9.13E-19 | 4.315 | Activated by Aged | Ap1, CBX3, CCND1, CDKN1A, CSF2, E2F1, E2F4, E2f, ERBB2, FOXM1, HDAC1, MYC, NFKBIA,NFkB (complex), RB1, RBL2, RELA, Rb, SP1, STAT3, TP53 |
| 12 | IFNL1 | 1.26E-13 | 4.284 | Activated by Aged | CEBPA, CREBBP, IFNL1, IRF9, JAK1, NFKB1, NFkB (complex), RELA, RNA polymerase II, STAT1, STAT2, STAT3, STAT4 |
| 13 | calcitriol | 1.53E-24 | 4.222 | Activated by Aged | CCND1, CDKN1A, CEBPB, E2F1, E2F4, E2f, ESR1, FOXO3, HDAC1, IL6, IRF1, MED1, NFkB (complex), PPARA, PPARG, RARA, RB1, RBL1, RXRA, Rb, SP1, STAT3, TBX2, THRB, TP53, calcitriol |
| 14 | IFNB | 2.37E-11 | 4.170 | Activated by Aged | CREBBP, IFN Beta, IFNG, IRF1, IRF7, IRF8, IRF9, Ifnar, NFkB (complex), RELA, STAT1, STAT2, STAT3, STAT4 |
| 15 | IRF3 | 1.71E-12 | 4.133 | Activated by Aged | CREBBP, IFN type 1, IFNA2, IFNB1, IRF1, IRF3, IRF7, IRF9, ISGF3, NFkB (complex), STAT1, STAT2, STAT3, STAT4 |
| 16 | IFNB1 | 1.11E-12 | 4.085 | Activated by Aged | CREBBP, IFNB1, IFNG, IRF1, IRF3, IRF7, IRF8, IRF9, Interferon alpha, NFkB (complex), STAT1, STAT2, STAT3 |
| 17 | doxorubicin | 1.09E-11 | 4.076 | Activated by Aged | CBX3, CCND1, CDKN1A, CDKN2A, E2F1, E2F2, E2F4, E2f, FOXM1, FOXO1, MYC, NFkB (complex), RB1, RBL2, Rb, TP53, doxorubicin |
|  |  |  |  |  |  |
| Rank | Upstream regulator | p-value | Activation z-score | Prediction activation state | Mechanistic Network |
| 4 | RABL6 | 5.88E-16 | -4.025 | Inhibited by Aged | None |
