# Supplementary material for: Age-associated mRNA expression changes in bovine endometrial cells in vitro
Source: Reprod Biol Endocrinol. 2017 Aug 14;15:63. doi: 10.1186/s12958-017-0284-z (PMC5556672; doi:10.1186/s12958-017-0284-z)
Supplement: Supplementary file 6 — Upregulated molecules by IFNT treatment in bovine aged endometrial cells and comparison with young endometrial cells. (DOCX 16 kb) [file 12958_2017_284_MOESM6_ESM.docx]

| Additional file 6: Table S6. Upregulated molecules by IFNT treatment in bovine aged endometrial cells and comparison with young endometrial cells | | | | | | | | | | | | | |
| --- | --- | --- | --- | --- | --- | --- | --- | --- | --- | --- | --- | --- | --- |
|  |  |  |  |  |  |  |  |  |  |  |  |  |  |
| Rank | Molecules | Aged Control RPKM | Aged IFNT RPKM | Fold change |  | Young Control RPKM | Young IFNT RPKM | Fold change |  |  |  |  |  |
| 1 | TPT1 | 5.55 | 19.58 | 3.53 |  | 24.94 | 16.25 | -1.53 |  |  |  |  |  |
| 2 | RPL17 | 4.58 | 12.14 | 2.65 |  | 12.64 | 2.31 | -5.47 |  |  |  |  |  |
| 3 | CXCL9 | 5.74 | 12.67 | 2.21 |  | 0.95 | 1.63 | 1.71 |  |  |  |  |  |
| 4 | CXCL10 | 9.70 | 19.51 | 2.01 |  | 1.46 | 2.01 | 1.38 |  |  |  |  |  |
